# Effects of Vitamin B_12_ Deficiency on Amyloid-β Toxicity in *Caenorhabditis elegans*

**DOI:** 10.3390/antiox10060962

**Published:** 2021-06-15

**Authors:** Arif Andra, Shoko Tanigawa, Tomohiro Bito, Atsushi Ishihara, Fumio Watanabe, Yukinori Yabuta

**Affiliations:** Department of Agricultural Science, Graduate School of Sustainability Science, Tottori University, 4-101 Koyama-Minami, Tottori 680-8553, Japan; arifandra94@gmail.com (A.A.); m21j7025x@edu.tottori-u.ac.jp (S.T.); bito@tottori-u.ac.jp (T.B.); aishihara@tottori-u.ac.jp (A.I.); watanabe@tottori-u.ac.jp (F.W.)

**Keywords:** Alzheimer’s disease, amyloid-β, *Caenorhabditis elegans*, oxidative stress, reactive oxygen species, vitamin B_12_

## Abstract

High homocysteine (Hcy) levels, mainly caused by vitamin B_12_ deficiency, have been reported to induce amyloid-β (Aβ) formation and tau hyperphosphorylation in mouse models of Alzheimer’s disease. However, the relationship between B_12_ deficiency and Aβ aggregation is poorly understood, as is the associated mechanism. In the current study, we used the transgenic *C. elegans* strain GMC101, which expresses human Aβ_1–42_ peptides in muscle cells, to investigate the effects of B_12_ deficiency on Aβ aggregation–associated paralysis. *C. elegans* GMC101 was grown on nematode growth medium with or without B_12_ supplementation or with 2-*O*-α-D-glucopyranosyl-L-ascorbic acid (AsA-2G) supplementation. The worms were age-synchronized by hypochlorite bleaching and incubated at 20 °C. After the worms reached the young adult stage, the temperature was increased to 25 °C to induce Aβ production. Worms lacking B_12_ supplementation exhibited paralysis faster and more severely than those that received it. Furthermore, supplementing B_12_-deficient growth medium with AsA-2G rescued the paralysis phenotype. However, AsA-2G had no effect on the aggregation of Aβ peptides. Our results indicated that B_12_ supplementation lowered Hcy levels and alleviated Aβ toxicity, suggesting that oxidative stress caused by elevated Hcy levels is an important factor in Aβ toxicity.

## 1. Introduction

B_12_ is a water-soluble vitamin that is well known for its complex chemical structure. The B_12_ molecule is composed of a corrin ring with a central cobalt atom linked to dimethylbenzimidazole. B_12_ exists in four different chemical forms, depending on the chemical group bound to the cobalt atom. Most commercially available B_12_ supplements contain a cyano group and are known as cyanocobalamin. In biological systems, the cyano group can be replaced with adenosyl, or methyl group to form the biologically active form adenosylcobalamin (AdoCbl), or methylcobalamin (MeCbl), respectively [1]. Humans mainly acquire B_12_ by consuming meat, dairy products, fish, and shellfish, as plants and mushrooms do not contain substantial amounts [2].

B_12_-protein complexes in food must be digested by pepsin in the stomach to enable absorption. Free B_12_ is transported by three different B_12_-binding proteins, haptocorrin, intrinsic factor, and transcobalamin, before being absorbed in the small intestine. Elderly people tend to have a lower rate of B_12_ absorption due to a high prevalence of atrophic gastritis [3], which decreases the production of pepsin and intrinsic factor, resulting in B_12_ malabsorption. Therefore, vegetarians and the elderly are the most prone to B_12_ deficiency.

B_12_ plays an important role in the production of red blood cells, synthesis of DNA, and protection of the nervous system [1,4]. The active forms, AdoCbl and MeCbl, function as cofactors for the enzymes methylmalonyl-CoA mutase (MCM, EC 5.4.99.2) and methionine synthase (MS, EC 2.1.1.13), respectively. MCM catalyzes the isomerization of L-methylmalonyl-CoA to succinyl-CoA, whereas MS catalyzes the synthesis of methionine from homocysteine (Hcy) [4]. B_12_ deficiency disrupts the homeostasis of propionate catabolism and the *S*-adenosyl methionine cycle, leading to the accumulation of Hcy and methylmalonic acid (MMA), a decrease in reduced glutathione levels, and a decrease in immune response control, resulting in oxidative stress [5]. Oxidative stress occurs when there is an imbalance between the production of reactive oxygen species (ROS) and the availability of antioxidants in the body. Furthermore, excessive production of ROS leads to the degradation of macromolecules, resulting in cellular damage that triggers various diseases such as diabetes, cardiovascular diseases, carcinogenesis, and neurodegeneration [5].

Alzheimer’s disease (AD) is the most common cause of dementia, accounting for 60–80% of all cases, and has been estimated to affect approximately 10–30% of people over 65 years old [6,7]. AD is characterized by the extracellular aggregation of Aβ and the accumulation of hyperphosphorylated tau protein in neurons (neurofibrillary tangles) [7]. Aβ peptide found in the brains of patients with AD is generated after consecutive cleavages of amyloid precursor protein (APP) by β- and γ-secretases [8]. Although AD has been studied for more than a century, owing to its complex mechanism, many clinical trials conducted to discover effective therapeutic strategies against AD have failed [9]. Current treatments for AD only treat the symptoms and are unable to prevent progression of the disease [7].

B_12_ deficiency has been proposed as a risk factor for AD [1]. In addition, elevated plasma Hcy levels, mainly caused by B_12_ deficiency, have been observed in elderly individuals with mild cognitive impairment [1]. Furthermore, a study using transgenic mouse models of AD reported that hyperhomocysteinemia causes memory deficits, increases Aβ peptide accumulation, and increases tau phosphorylation—the three major pathological features of AD [10]. However, whether a high plasma Hcy level is the primary factor in AD pathology in humans or merely a marker for another underlying condition, such as low folate level, poor lifestyle, or renal failure, is still under debate [11]. In addition, other consequences of B_12_ deficiency, such as mitochondrial dysfunction and oxidative stress [5], must also be considered to understand the relationship between high plasma Hcy levels and the mechanism of AD.

*Caenorhabditis elegans* is a powerful genetic model organism that was introduced by Sydney Brenner [12]. This free-living roundworm has been widely used as an animal model in various fields of study, including neurodegenerative disease research. In our previous study, we successfully induced B_12_ deficiency in *C. elegans* by growing them in M9 minimal media with *Escherichia coli* OP50 as a food source [13]. Worms fed a strict B_12_-restricted diet exhibited elevated Hcy and MMA levels, fertility loss, extended life cycles, and reduced lifespans. In addition, *C. elegans* grown on nematode growth medium (NGM) with *E. coli* OP50 as a food source have also been reported to exhibit B_12_ deficiency [14].

Transgenic *C. elegans* worms expressing Aβ peptides have been engineered to mimic the pathological features of AD [15]. Multiple strains of transgenic worms have been developed to produce Aβ peptides. In the *C. elegans* strain, GMC101, the production of Aβ_1–42_ peptides in muscle cells is induced by temperature upshift [16]. In the current study, we used the GMC101 strain to investigate the effects of B_12_ deficiency on Aβ peptide toxicity and to gain a better understanding of the relationship between B_12_ deficiency and AD.

## 2. Materials and Methods

### 2.1. C. elegans Strains

*Pacdh-1::GFP* (VL749) transgenic worms were used as reporters of dietary B_12_ status [14]. The VL749 and GMC101 strains used in the current study were provided by the Caenorhabditis Genetic Center (CGC), funded by the NIH Office of Research Infrastructure Programs (P40 OD010440). The worms were maintained at 20 °C.

### 2.2. Preparation of B_12_-Deficient Worms

Worms grown on NGM (1 mmol/L MgSO_4_, 1 mmol/L CaCl_2_, 5 mg/L cholesterol, 25 mmol/L KPO_4_ (pH 6), 17 g/L agar, 3 g/L NaCl, and 2.5 g/L bacto-peptone) without cyanocobalamin were considered B_12_ deficient. Worms administered B_12_ supplementation were grown on NGM with 100 μg/L cyanocobalamin.

### 2.3. Fluorescence Imaging of Pacdh: GFP Transgenic Worms

Worms were age-synchronized by hypochlorite bleaching and grown at 20 °C on NGM with or without B_12_ supplementation until they reached the young adult stage. The worms were then mounted on 5% agarose gel, and fluorescence images were captured using an SZX-RFL-2 fluorescence microscope (Olympus Co., Tokyo, Japan).

### 2.4. Measurement of B_12_-Related Biomarkers Using Liquid Chromatography–Mass Spectrometry (LC-MS/MS)

The N2 Bristol *C.* worms were age-synchronized by hypochlorite bleaching and grown at 20 °C on NGM with or without B_12_ supplementation until they reached the young adult stage. Then, the worms were collected from the plates and washed using M9 buffer. The worm pellets were then homogenized, and the supernatant was used for the measurement of Hcy and MMA levels via LC-MS/MS according to the methods described by Weaving et al. [17] and Mineva et al. [18], respectively.

### 2.5. Paralysis Assay

The paralysis assay was performed using GMC101 transgenic worms expressing Aβ peptide according to the method described by McColl et al. [16]. Briefly, *C. elegans* GMC101 was grown on NGM with or without B_12_ supplementation or with AsA-2G supplementation. The worms were age-synchronized by hypochlorite bleaching and incubated at 20 °C. After the worms reached the young adult stage, the temperature was increased to 25 °C to induce Aβ production. The number of paralyzed worms was counted at 12 h intervals. Worms unable to perform full-body wave propagation were scored as paralyzed.

### 2.6. Quantitative Reverse Transcription PCR (qRT-PCR)

GMC101 worms were age-synchronized by hypochlorite bleaching and grown at 20 °C on NGM with or without B_12_ supplementation until they reached the young adult stage. Worms were then transferred to NGM plates containing 75 μmol/L 5-fluoro-2′-deoxyuridine, and the temperature was increased to 25 °C to induce Aβ peptide expression. After 24 h, worms were collected, snap-frozen in liquid nitrogen, and stored at -80 °C until RNA extraction was performed using Sephasol^®^-RNA1 (Nacalai Tesque, Kyoto, Japan). Total RNA was used for cDNA synthesis using the PrimeScript™ RT Reagent Kit with gDNA Eraser (Takara Bio, Shiga, Japan). The following primers were used for the amplification: *actin-1* (T04C12.6) (F) 5′-TCCAAGAGAGGTATCCTTACCC-3′ and (R) 5′-CTCCATATCATCCCAGTTGGTG-3′; Aβ (F) 5′-GCGGATGCAGAATTCCGACATGAC-3′ and (R) 5′-TATGACAACACCGCCCACCATGAG-3′. qPCR was performed on a CFX Connect Real-Time System (Bio-Rad Laboratories, Inc., Berkeley, CA, USA) using GeneAce SYBR^®^ qPCR Mix α (Nippon Gene Co., Ltd., Tokyo, Japan). The mRNA level of Aβ was normalized to that of *actin-1*.

### 2.7. In Situ Detection of Intracellular ROS

Intracellular ROS production was detected via 2′,7′-dichlorodihydrofluorescein diacetate (DCFH-DA) assay, as previously described [19]. Briefly, N2 worms were age-synchronized by hypochlorite bleaching and grown at 20 °C on NGM with or without B_12_ supplementation until they reached the young adult stage. The worms were then collected from the plates and washed with M9 buffer. DCFH-DA was dissolved in dimethyl sulfoxide, diluted to a final concentration of 100 μM using sterilized M9 medium, and used as a staining solution. Worms grown with or without B_12_ supplementation (labeled B_12_^+^ and B_12_^−^, respectively; approximately 12 worms per group) were treated with 1 mL of the staining solution for 5 h in the dark. Following the staining, the worms were washed 10 times with sterile water and mounted on 5% agarose gel. Fluorescence images were captured using the BZ-9000 series HS All-in-One fluorescence microscope with an FITC filter (Keyence, Osaka, Japan). The anterior part of the worms was observed. Exposure time was 666 ms. Fluorescence intensity was quantified using ImageJ (ImageJ Software, Bethesda, MD, USA, http://imagej.nih.gov/ij/, accessed on 18 May 2021) for 8 worms per treatment condition.

### 2.8. Immunoblot Analysis

Immunoblot analysis was performed as previously described [20], with a few modifications. GMC101 worms were weighed and homogenized in 500 µL of phosphate-buffered saline. The homogenates were centrifuged at 800× *g* for 10 min, and the supernatants were subjected to sodium dodecyl sulfate polyacrylamide gel electrophoresis (SDS–PAGE) and immunoblotting. SDS–PAGE was performed using p-PAGEL slab gels (P–T16.5S; ATTO Corporation, Tokyo, Japan) according to the manufacturer’s instructions.

The gels were then stained with Coomassie Brilliant Blue R-250, and proteins were transferred to polyvinylidene difluoride membranes (Immuno-Blot PVDF; Bio-Rad Laboratories, Hercules, CA, USA) using an electroblotting apparatus (model 200/2.0, Bio-Rad Laboratories) set at 13 V for 30 min. Aβ peptide was detected using a monoclonal anti-Aβ_1–16_ primary antibody (BioLegend, San Diego, CA, USA) and an anti-mouse IgG–horseradish peroxidase conjugate (Promega Corp. Madison, WI, USA) secondary antibody. Tubulin (the loading control) was detected using a monoclonal anti-tubulin primary antibody (ab6160, Abcam, Cambridge, MA, USA) and an anti-mouse IgG–horseradish peroxidase conjugate (SA00001-15, Proteintech Japan, Tokyo, Japan) secondary antibody. Signals were detected using EzWestBlue (ATTO Corporation) according to the manufacturer’s instructions.

### 2.9. Statistical Analyses

Statistical differences were assessed using the two-tailed Student’s *t*-test with parametric unequal variance in Microsoft Excel 2013 (Microsoft, Redmond, WA, USA). Data in graphs are presented as the mean ± standard deviation (SD). Values of *p* < 0.05 were considered statistically significant.

## 3. Results

### 3.1. Worms Grown on NGM without B_12_ Supplementation Exhibited B_12_ Deficiency

Although it was possible to induce severe B_12_ deficiency in worms by growing them on M9 medium without B_12_ supplementation for five generations, the limited amount of nutrition available in M9 medium may have inhibited Aβ expression. Furthermore, Revtovich et al. [21] reported that worms grown on M9 medium for five generations have lower fecundity, suggesting that M9 medium has insufficient nutrition to support the development of worms. Therefore, since worms grown on NGM with *E. coli* OP50 as a food source have been reported to exhibit B_12_ deficiency [14,21], we used NGM with or without B_12_ supplementation for the paralysis assay.

**Figure 1:**
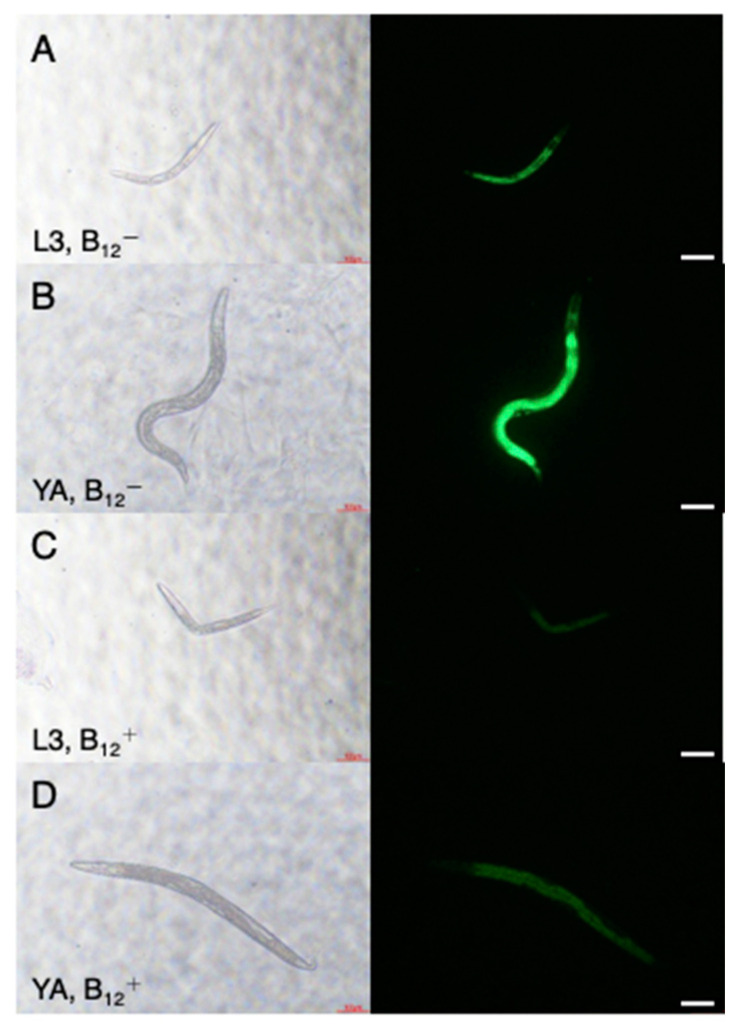
Representative fluorescence images of L3 and young adult VL749 B_12_^−^ and B_12_^+^ worms. (**A**), L3 B_12_^−^ larva; (**B**), young adult B_12_^−^ worm; (**C**), L3 B_12_^+^ larva; (**D**), young adult B_12_^+^ worm. Worms grown with B_12_ supplementation, B_12_^+^; without B_12_ supplementation, B_12_^−^. Scale bars = 100 μm.

**Figure 2:**
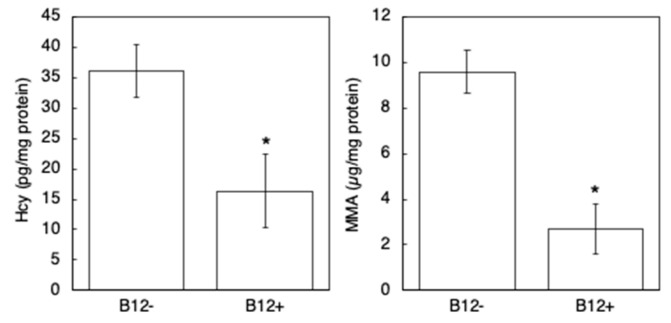
Hcy and MMA levels in B_12_^−^ and B_12_^+^ N2 worms. Hcy and MMA levels were measured as described in the Materials and Methods section. All values represent the mean ± SD of three independent experiments (n = 3). Asterisks indicate significant differences compared to the B_12_^−^ worms (* *p* < 0.05).

Before performing paralysis assay in this culture condition, first, fluorescence images of *Pacdh-1::GFP* (VL749) worms were taken to observe dietary B_12_ status of worms cultivated on NGM with or without B_12_ supplementation (B_12_^+^ worms or B_12_^−^ worms). The B_12_^−^ VL749 worms exhibited GFP expression, which increased as the worms developed (Figure 1A,B), whereas no GFP expression was observed in the B_12_^+^ VL749 worms (Figure 1C,D). Furthermore, to investigate the degree of B_12_ deficiency in the B_12_^−^ worms, Hcy and MMA levels were measured using LC-MS/MS. The results showed a significant accumulation of Hcy and MMA in B_12_^−^ worms compared to that in B_12_^+^ worms (Figure 2); the Hcy and MMA levels in the B_12_^−^ worms were 2.2- and 3.6-fold higher, respectively, than those in the B_12_^+^ worms. These findings clearly indicated the B_12_ deficiency in the B_12_^−^ worms.

### 3.2. B_12_ Supplementation Reduced Aβ Toxicity in B_12_-Deficient GMC101 Worms

**Figure 3:**
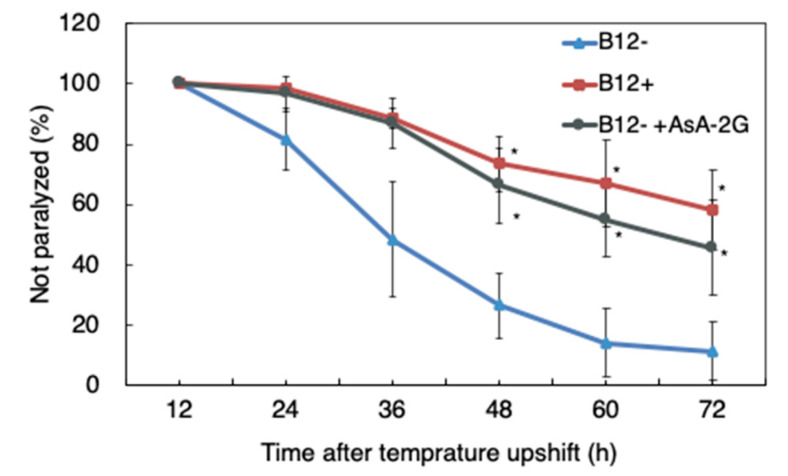
Paralysis rate of B_12_^−^, B_12_^+^, and B_12_^−^ +AsA-2G GMC101 worms. Young adult worms were incubated at 25 °C to induce Aβ production. The mean percentage of unparalyzed worms is plotted against the time post temperature shift (h). All values represent the mean ± SD of three independent experiments (n = 3). Approximately 270 worms were screened for each condition. Asterisks indicate significant differences compared to the B_12_^−^ worms at same time point (* *p* < 0.05).

To evaluate the effects of B_12_ deficiency on the nervous system, we performed a paralysis assay using GMC101 worms grown on NGM with or without B_12_ supplementation. After 48 h of incubation, approximately 74% of the B_12_^−^ GMC101 worms were paralyzed, whereas after 72 h, more than 90% of the worms were paralyzed (Figure 3). In contrast, only 26% of the B_12_^+^ GMC101 worms were paralyzed after 48 h. The paralysis rate was found to be significantly different between the B_12_^+^ and B_12_^−^ GMC101 worms 48 h after the temperature upshift.

**Figure 4:**
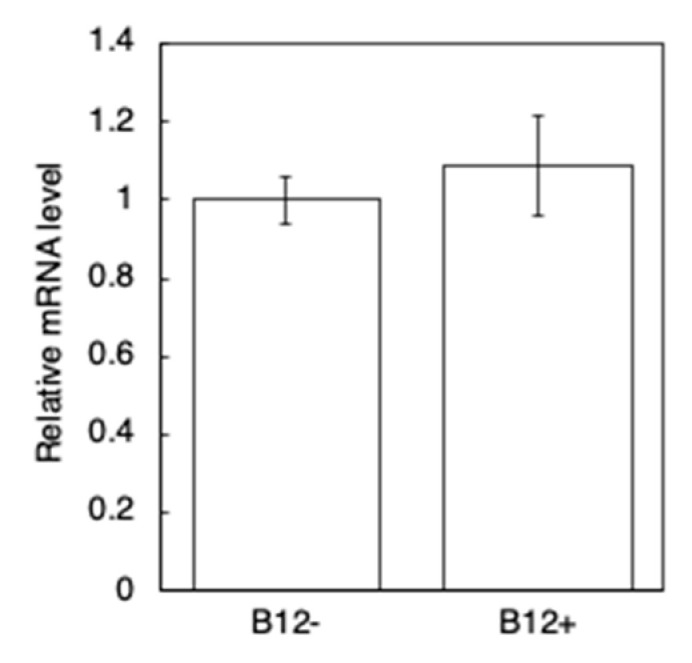
Aβ mRNA levels in B_12_^−^, and B_12_^+^ GMC101 worms. The relative expression of Aβ mRNA was normalized to that of *actin-1*. The expression value for B_12_^−^ worms was set to 1. The data represent the mean ± SD of three independent experiments (n = 3).

**Figure 5:**
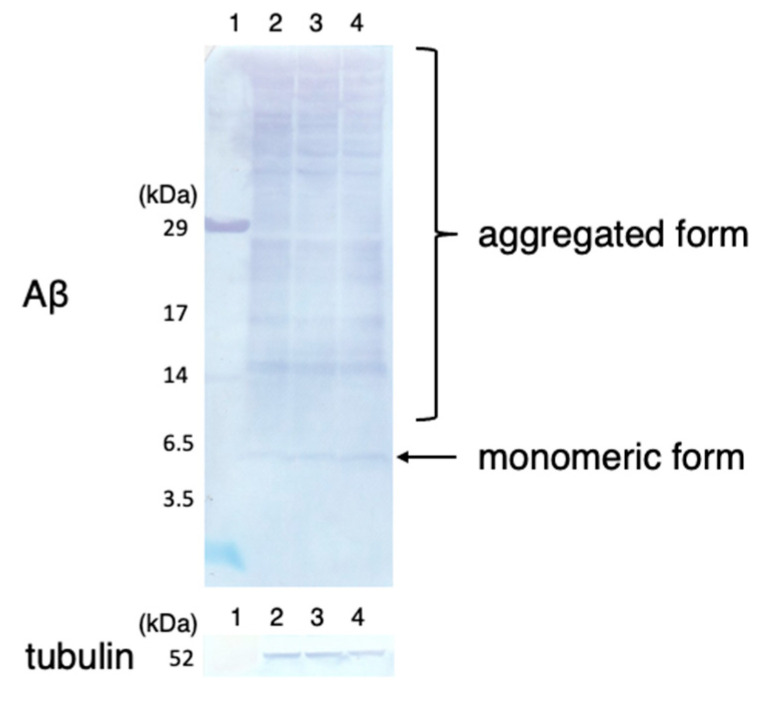
Accumulation of Aβ peptides in B_12_^−^, B_12_^+^, and B_12_^−^ +AsA-2G GMC101 worms. The crude extract from B_12_^−^, B_12_^+^, or B_12_^−^ +AsA-2G worms (40 µg each) was subjected to SDS-PAGE and immunoblotting using antibodies against anti-β-amyloid(_1–16_) and anti-tubulin antibodies. Lane 1, molecular marker; lane 2, B_12_^+^ worms; lane 3, B_12_^−^ worms; lane 4, B_12_^−^ +AsA-2G worms.

To investigate if B_12_ supplementation affects the transcription level of Aβ rather than simply reducing its accumulation in muscle cells, qRT-PCR of the B_12_^+^ and B_12_^−^ GMC101 worms was performed 24 h after inducing Aβ expression. The results indicated no significant difference in the Aβ mRNA levels between the B_12_^+^ and B_12_^−^ GMC101 worms (Figure 4). Additionally, immunoblotting indicated no significant difference in the level of the monomeric form and aggregated form of the Aβ peptide (Aβ_1–42_) between the B_12_^−^ and B_12_^+^ GMC101 worms (Figure 5).

### 3.3. NGM Supplemented with AsA-2G Ameliorated Aβ Toxicity in B_12_-Deficient GMC101 Worms

**Figure 6:**
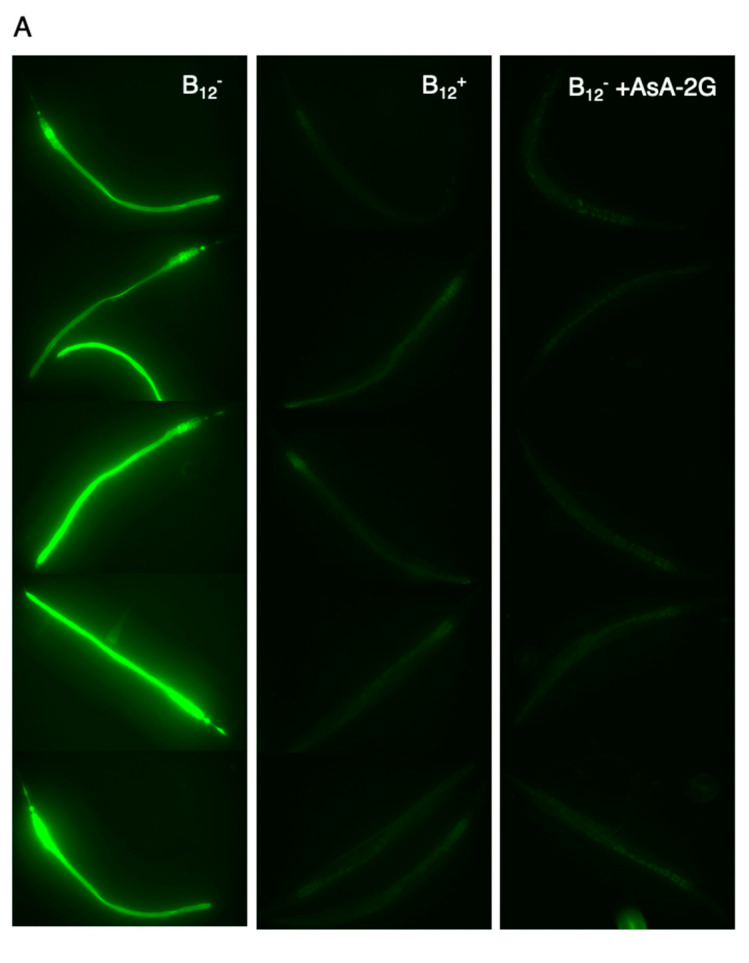

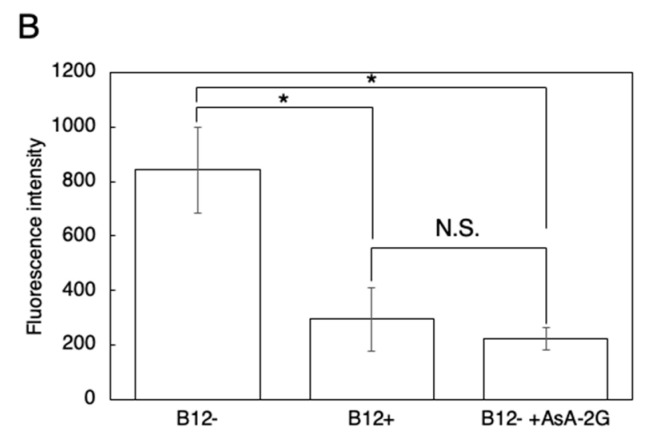
ROS levels in B_12_^−^, B_12_^+^, and B_12_^−^ +AsA-2G N2 worms. (**A**). Fluorescence images of young adult N2 B_12_^+^ or B_12_^−^ worms. Reactive oxygen species (ROS) accumulation in the worms was assessed via assay of the ROS indicator 2′,7′-dichlorodihydrofluorescein diacetate. (**B**). Fluorescence intensity. All values represent the mean ± SD of eight worms per treatment condition (n = 8). Asterisks indicate significant differences (* *p* < 0.05). N.S., not significant.

Oxidative stress induced by B_12_ deficiency may have enhanced the paralysis rate of B_12_^−^ GMC101 worms [22]. Therefore, we determined the amount of ROS accumulation in B_12_^+^ and B_12_^−^ worms using DCFH-DA, an indicator of ROS. We observed a high level of ROS accumulation in the B_12_^−^ worms compared to that in B_12_^+^ worms (Figure 6), which supported the findings from a recent study [22].

To investigate the effect of reduced ROS levels in B_12_^−^ GMC101 worms on paralysis and aggregation of the Aβ peptide, the B_12_^−^ worms were supplemented with AsA-2G, a stable ascorbic acid. Supplementation with AsA-2G reduced the amount of ROS accumulation in B_12_^−^ worms to the same level as in B_12_^+^ worms (Figure 6). Interestingly, AsA-2G supplementation improved the paralysis phenotype in the B_12_^−^ GMC101 worms (B_12_^−^ +AsA-2G) (Figure 3). However, AsA-2G supplementation did not affect to aggregation of the Aβ peptide (Figure 5).

## 4. Discussion

AD is a progressive neurodegenerative disorder characterized by memory impairment, disorientation, behavioral changes, and impaired communication [6]. A high Hcy level, caused by low B_12_ or folate levels, is known to be one of the risk factors for AD [11]. In a clinical trial conducted on healthy elderly individuals with high Hcy levels, folate supplementation over three years improved global functioning, memory storage, and information-processing speed [23], suggesting that AD may be prevented by lowering Hcy levels. In the current study, we used a transgenic *C. elegans* model expressing the Aβ_1–42_ peptide in muscle cells to investigate the effect of B_12_ deficiency on Aβ toxicity. Our results showed that the level of Hcy and MMA accumulation was higher in B_12_^−^ worms than in B_12_^+^ worms (Figure 2). In addition, B_12_^−^ GMC101 worms exhibited a higher paralysis rate (Figure 3) and higher ROS levels (Figure 6) than B_12_^+^ worms.

In addition to its primary function as a cofactor, B_12_ is known to possess antioxidant properties. Chan et al. [24] demonstrated that B_12_ alleviates cellular oxidative stress by directly scavenging superoxide, in vitro and in vivo. Additionally, the protective properties of B_12_ include the preservation of glutathione and modulation of cytokines and growth factors that induce immune response–mediated oxidative stress [5]. Furthermore, Hcy is known to produce ROS by autoxidation [25] and activation of NADPH oxidase [26], although the complete mechanism is unclear. In addition, MMA is known to induce oxidative stress [27]. Thus, B_12_ deficiency induces oxidative stress through various mechanisms.

To investigate the effects of oxidative stress caused by B_12_ deficiency on Aβ toxicity, B_12_^−^ GMC101 worms were grown with AsA-2G supplementation. As expected, AsA-2G supplementation alleviated paralysis, and decreased ROS levels in B_12_^−^ worms (Figure 3 and Figure 6). However, AsA-2G supplementation had no effect on the aggregation of Aβ peptides, suggesting that the enhanced ROS level was not involved in promoting Aβ aggregation.

Yatin et al. [28] reported that Aβ_1–42_ peptide alone produces free radicals in vitro and that Aβ peptide causes oxidative stress in rat neuron cells and *C. elegans*. These findings suggest that Aβ toxicity is associated with oxidative stress. Interestingly, oxidative stress and the paralysis phenotype in the transgenic *C. elegans* preceded the fibrillar deposition of Aβ peptides, suggesting that Aβ toxicity is caused by pre-fibrillar Aβ [29]. Multiple studies have reported that increased oxidative stress plays an important role in the pathogenesis of AD; oxidative stress can accelerate Aβ generation and aggregation, tau hyperphosphorylation and aggregation, and neuronal apoptosis (reviewed by Wu et al. [30]). As tau hyperphosphorylation and aggregation and neuronal apoptosis were not observed in the GMC101 worms, our results suggest that oxidative stress induced by B_12_ deficiency may be associated with the cellular events leading to paralysis after Aβ expression. In conclusion, oxidative stress caused by elevated Hcy and/or MMA levels may enhance Aβ toxicity in B_12_-deficient worms.

Transcriptomic analysis has been performed to elucidate the mechanisms associated with paralysis caused by Aβ expression [31]; however, these mechanisms are not completely understood. Enhanced mitochondrial proteostasis has been reported to reduce the amount of Aβ aggregation in cells, GMC101 worms, and transgenic mouse models of AD [32]. Further analyses are required to understand the molecular mechanisms underlying the alleviation of Aβ toxicity by AsA-2G supplementation.

B_12_ has a relatively low recommended dietary allowance compared to other micronutrients, and our body maintains sufficient storage of B_12_ for up to several years. Therefore, clinical B_12_ deficiency is uncommon and is mainly found in patients with hereditary diseases [4]. However, with age, the risk of developing B_12_ deficiency increases due to several factors, such as malabsorption and depletion of B_12_ storage [4]. A high Hcy level caused by B_12_ deficiency is a known risk factor for neurodegenerative diseases; approximately 20% of dementia cases are reported to be strongly related to high plasma Hcy levels [33]. Furthermore, high Hcy and MMA levels have been observed in elderly people with normal or high serum B_12_ levels [34]. Oxidative stress is known to promote the generation and aggregation of Aβ, which is believed to be a toxic factor linked to AD pathogenesis [30]. Therefore, a higher daily intake of B_12_, folate, and antioxidants is required to lower Hcy and MMA levels and reduce the incidence of AD.

In conclusion, our study suggests that the oxidative stress induced by B_12_ deficiency promotes Aβ toxicity. Although the detailed mechanism is unknown, we will investigate the effect of B_12_ deficiency on mitochondrial proteostasis.

## Data Availability

The data supporting the results are available from the corresponding author on reasonable request.
